# Modulation of microglial activation by antidepressants

**DOI:** 10.1177/02698811211069110

**Published:** 2022-01-29

**Authors:** Nicole Mariani, James Everson, Carmine M Pariante, Alessandra Borsini

**Affiliations:** Stress, Psychiatry and Immunology Laboratory, Department of Psychological Medicine, Institute of Psychiatry, Psychology & Neuroscience, King’s College London, London, UK

**Keywords:** Microglia, antidepressants, neuroinflammation, depression

## Abstract

**Background:**

Recent studies have suggested that microglial activation plays a key role in the pathogenesis of depression. In fact, neuroinflammation is associated with a phenotypic change of microglia, consisting of morphological differences, increased release of cytokines and oxidative stress products, which may contribute to the development and maintenance of depression. Antidepressants, including selective serotonin re-uptake inhibitors and serotonin–norepinephrine reuptake inhibitors, have been shown to act on the immune and oxidative stress mechanisms commonly found to be disrupted in depression. Thus, the inhibition of microglial activation may be one of the mechanisms through which they exert an antidepressant action.

**Aim:**

This is the first review summarising in vitro and ex vivo studies investigating the effects of different classes of antidepressants on microglia activation, by examining cellular changes and/or via measuring the production of immune and/or oxidative stress signalling molecules, in microglia models of neuroinflammation with either lipopolysaccharide (LPS) or cytokines. A total of 23 studies were identified, 18 using LPS stimulation and 5 using cytokines stimulation.

**Results:**

Overall, the studies show that antidepressants, such as selective serotonin re-uptake inhibitors, serotonin–norepinephrine reuptake inhibitors, monoamine oxidase inhibitors and tricyclic antidepressants prevented microglial activation, including reduced microglial reactivity and decreased immune and oxidative stress products, in both models. However, specific antidepressants, such as bupropion and agomelatine did not prevent interferon-gamma (IFN-γ)-induced microglial activation; and for other antidepressants, including phenelzine, venlafaxine and sertraline, the results of different studies were inconsistent.

**Conclusions:**

Overall, results summarised in this review support the hypothesis that the action of at least certain classes of antidepressants may involve regulation of microglial activation, especially when in presence of increased levels of inflammation.

## Introduction

Major depressive disorder (MDD) is projected to be the leading cause of disease burden globally by 2030 (World Health Organization, 2011). The focus of depressive pathophysiology has evolved from dysregulated monoaminergic neurotransmission towards increased neuroinflammation, affecting both neuronal and glial function under the environmental influence (Abbink et al., 2019; van den Bosch and Meyer-Lindenberg, 2019). In fact, it is clear that dysfunctions in excitatory and/or inhibitory glutamate and gamma-aminobutyric acid (GABA) signalling mechanisms may play a critical role in depression (Fee et al., 2017; Goshen et al., 2008; Luscher and Fuchs, 2015; Luscher et al., 2011). It is also well-established that inflammation within the central nervous system (CNS) significantly contributes to the development of psychiatric diseases (Stephenson et al., 2018), whereby pro-inflammatory cytokines, such as interleukin 1 beta (IL-1β), IL-6 and tumour necrosis factor-alpha (TNF-α) are significantly raised in serum and cerebral spinal fluid (CSF) of, at least, a subgroup of patients suffering from depression (Wang and Miller, 2018; Zou et al., 2018). Indeed, within the CNS, cytokines are secreted by numerous cell populations, including neurons and astrocytes (Galic et al., 2012), however, since microglia act as the principal immune cells in the brain, they play a central role in regulating distinct neuroinflammatory processes involved in the depressive psychopathology (Perry and Teeling, 2013; Réus et al., 2015). To support this evidence, different studies have shown that a high proportion of cancer and hepatitis C patients who receive cytokine including interferon-alpha (IFN-α) therapy develops symptoms of depression that are indistinguishable from those found in MDDs (Denicoff et al., 1987; Schäfer et al., 2007; Su et al., 2010; Udina et al., 2012; Valentine et al., 1998). In fact, IFN-α treatment can lead to the development of depression in up to 30% of patients within the first 3 months of therapy (Schaefer et al., 2012). IFN-α is a cytokine associated with an early viral infection and has both anti-viral and anti-proliferative properties (Stark et al., 1998).

Several pre-clinical (Lehmann et al., 2016; Wang et al., 2018b) and clinical studies in depression, including neuroimaging (Holmes et al., 2018) and post-mortem studies (Schnieder et al., 2014; Steiner et al., 2008, 2011), have shown an increase in the numbers of activated microglia, in particular, in individuals with suicidal ideation. Specifically, neuroimaging studies have analysed microglial activation through measurement of positron emission tomography (PET) radioligands targeting translocator protein (TSPO), a protein located on outer mitochondrial membranes in microglia. In contrast, post-mortem studies, have investigated microglial reactivity through other markers, including major histocompatibility complex class II (MHC-II), CD40, CD68 and the peripheral-type benzodiazepine receptor. In particular, expression of MHC-II (i.e. HLA-DR) in microglial cells is often an indication of activated neuroinflammatory and neurodegenerative processes in histological studies and, therefore, considered a reliable marker of microglial reactivity (Schmitt et al., 1998).

Microglia naturally exist within the M2 phenotype, mediating synaptic pruning and surveilling the CNS parenchyma to maintain homeostasis (Nimmerjahn et al., 2005). However, biological or psychosocial insults, such as infections or periods of chronic stress, stimulate microglia to enter the M1 phenotype, where surveillance and synaptic pruning are reduced, and further inflammation is encouraged, through an increase in pro-inflammatory cytokines, such as IL-1β, IL-6 and TNF-α (Zhao et al., 2019). However, it is reductive to classify microglia’s phenotypes in a similar way to peripheral macrophages. In fact, according to findings from genetic data analyses (Martinez and Gordon, 2014; Ransohoff, 2016), this classification method needs reconsideration of the restrictive M1/M2 activation paradigm to fully describe the broad diversity of microglial response. Specifically, the numerous functions of microglia, integrating both immune and metabolic cell characteristics, would be achieved by obtaining multiple phenotypes, each of which is associated with a different molecular signature (Bennett et al., 2016; Crain et al., 2013; Hammond et al., 2019; Hickman et al., 2013). Said that, while microglia exist on a spectrum of polarisation, it is well-established that neuroinflammation is associated with a phenotypic change of microglia. This phenotypic change may occur due to morphological differences, as well as by the increased release of cytokines and oxidative stress products (Harry and Kraft, 2008; Lehnardt, 2010), which can ultimately disrupt neuronal differentiation and increase cell death, similar to what is often observed in pre-clinical (Goshen et al., 2008; Koo and Duman, 2008) and post-mortem studies in depression (Boldrini et al., 2013).

Interestingly, a few classes of antidepressants, including selective serotonin re-uptake inhibitors (SSRIs) and serotonin–norepinephrine reuptake inhibitors (SNRIs) can act on the same inflammatory and oxidative stress mechanisms commonly found to be disrupted in depression, and this may be one of the mechanisms through which they exert an antidepressant action. In fact, antidepressants can reduce inflammation and oxidative stress, as shown both in pre-clinical (Abdel-Salam et al., 2011; Hashioka, 2011; Myint et al., 2007) and clinical models of neuropsychiatric conditions (Hamer et al., 2011; Hashioka et al., 2009). However, the way antidepressants regulate these biological mechanisms remains unknown. Considering the fundamental role of microglia in regulating inflammation and oxidative stress signals on one hand (Harry and Kraft, 2008; Lehnardt, 2010), and the ability of antidepressants to govern microglial cell function on the other (Sanacora and Banasr, 2013), there is now a growing belief that one way for antidepressants to work is to inhibit microglial activation and ultimately reduce inflammation in the brain (Kalkman and Feuerbach, 2016).

Although the effect of different classes of antidepressant on neurons and astrocytes is well-documented in other reviews (Czéh and Di Benedetto, 2013; Drzyzga et al., 2009; Hanson et al., 2011), articles discussing their influence on microglial function in pre-clinical models of neuroinflammation is sparse, suggesting a need for a microglia-centric review. Although a previous review by Kalkman et al. in 2016 has discussed the anti-inflammatory effect of antidepressants on microglia/macrophage M1 polarisation, they have only reported studies on cells exposed to selected classes of antidepressants (SSRIs, monoamine oxidase inhibitors (MAOIs) and tricyclic antidepressants (TCAs)) where only inflammation, but not oxidative stress, has been investigated (Kalkman and Feuerbach, 2016). Hence, to our knowledge, this is the first and most updated review summarising all available findings on the effects of multiple classes of antidepressants, including SSRIs, SNRIs, TCAs, MAOIs and atypical antidepressants, on microglial activation, in lipopolysaccharide (LPS) and cytokines models of neuroinflammation. In particular, this narrative review collates in vitro and ex vivo studies, which investigated microglial activation, by assessing cellular changes and/or via measuring the production of inflammatory and/or oxidative stress signalling molecules, in microglia exposed to treatment with LPS/cytokines alone or in combination with an antidepressant. A total of 23 studies were identified, including 18 using stimulation with LPS and 5 using stimulation with cytokines. Studies were excluded if they did not measure at least one of our three outcomes (microglia morphological changes, inflammation or oxidative stress), did not use a direct biological challenge to model neuroinflammatory disease, were not in the English language, analysed homogenised brain tissue rather than just microglia, did not specify the analysed tissue or did not report the effect of an antidepressant on microglia directly.

## Results

### LPS-induced models of neuroinflammation

This section summarises the findings of 18 articles, which report the effect of SSRIs, SNRIs, tricyclic and MAOIs antidepressants in preventing microglial activation induced by LPS treatment in in vitro and ex vivo models of neuroinflammation. LPS administration is frequently used in several models of inflammation, both in vivo and in vitro models (Carvey et al., 2003; Gao et al., 2002). It has several essential advantages, including technical ease and high reproducibility, particularly in the inflammatory response produced. Across these studies, microglial activation has been identified by cellular changes and/or by the presence of inflammatory and/or oxidative stress products typical of an activated status.

#### Selective serotonin re-uptake inhibitors

##### Fluoxetine

Nine in vitro and three ex vivo studies investigated the effect of fluoxetine in preventing LPS-induced microglial activation (Table 1). Overall, studies showed that fluoxetine can prevent microglial cellular changes, as well as immune activation and oxidative stress production upon in vitro and ex vivo exposure to LPS.

**Table 1.** Studies examining the effects of antidepressants in preventing microglial activation induced by LPS treatment in in vitro and ex vivo models of neuroinflammation.

Article	Investigated tissue	Drug concentration	Duration	LPS concentration	Microglial activation outcomes					
					Cellular modulation		Inflammation		Oxidative stress	
					LPS (vs control)	LPS + Antidepressant (vs LPS)	LPS (vs control)	LPS + Antidepressant (vs LPS)	LPS (vs control)	LPS + Antidepressant (vs LPS)
SSRI antidepressants										
Fluoxetine										
Dhami et al. (2013)	In vitro: Sprague–Dawley rat primary microglia	10 μM	24 h	1 μg/mL	–	–	↑IL-1β, ↑TNF-α	↓IL-1β, ↓TNF-α	↑NO	↓NO
Du et al. (2014)	In vitro: C57BL/6 mice primary microglia	0.1 μM	24 h	100 ng/mL	–	–	↑TNF-α, ↑IL-6	↓TNF-α, ↓IL-6	↑NO	↓NO
		1 μM	24 h	100 ng/mL	–	–	↑TNF-α, ↑IL-6	↓TNF-α, ↓IL-6	↑NO	↓NO
		10 μM	24 h	100 ng/mL	–	–	↑TNF-α, ↑IL-6, ↑p-TAK1, ↓IkB, ↑NF-κB p65, ↑ β-Arrestin2, ↑TAK1-TAB1 interaction, ↓ β-Arrestin2-TAB1 interaction	↓TNF-α, ↓IL-6, ↓p-TAK1, ↑IkB, ↓NF-κB p65, ↓β-Arrestin2, ↓TAK1-TAB1 interaction, ↑β-Arrestin2-TAB1 interaction	↑NO	↓NO
	In vitro: β-arrestin2 knock-down C57BL/6 mice primary microglia	10 μM	24 h	100 ng/mL			↑TNF-α, ↑IL-6, ↑p-TAK1, ↓IkB, ↑NF-κB p65	= TNF-α, = IL-6, = p-TAK1, =IkB, = NF-κB p65	↑NO	= NO
Du et al. (2016)	In vitro: C57BL/6 mice primary microglia	0.1 μM	6 h	100 ng/mL (+5 mM ATP)	–	–	↑NLRP3, ↑IL-1β, ↑Pro-IL-1β	= NLRP3, ↓IL-1β, = Pro-IL-1β	–	–
		1 μM	6 h	100 ng/mL (+5 mM ATP)	–	–	↑NLRP3, ↑IL-1β, ↑Pro-IL-1β	↓NLRP3, ↓IL-1β, ↓Pro-IL-1β	–	–
		10 μM	6 h	100 ng/mL (+5 mM ATP)	–	–	↑NLRP3, ↑IL-1β, ↑Pro-IL-1β	↓NLRP3, ↓IL-1β, ↓Pro-IL-1β	–	–
		100 μM	6 h	100 μg/mL	–	–	–	–	↑NO	↓NO
Zhang et al. (2012)	In vitro: Fischer 344 rat primary microglia	3 μM	30 min	10 ng/mL	↑ Iba1	↓ Iba1	–	–	–	–
Liu et al. (2011)	In vitro: BV2 mice microglia	0.1 μM	24 h	100 ng/mL	–	–	↑TNF-α, ↑IL-6	↓TNF-α, = IL-6	↑NO	↓NO
		1 μM	24 h	100 ng/mL	–	–	↑TNF-α, ↑IL-6	↓TNF-α, ↓IL-6	↑NO	↓NO
		10 μM	24 h	100 ng/mL	–	–	↑TNF-α, ↑IL-6, ↑TNF-α mRNA, ↑IL-6 mRNA, ↑ERK1/2, ↑JNK, ↑p38 MAPK, ↓IkB-a, ↑NF-κB p65	↓TNF-α, ↓IL-6, ↓TNF-α mRNA, ↓IL-6 mRNA, = ERK1/2, = JNK, ↓p38 MAPK, ↑IkB-a, ↓NF-κB p65	↑NO, ↑iNOS, ↑iNOS mRNA	↓NO, ↓iNOS, ↓iNOS mRNA
	In vitro: BALB/c mouse; primary microglia	10 μM	24 h	100 ng/mL	↑CD11b	↓CD11b	↑p38 MAPK	↓p38 MAPK	↑iNOS	↓iNOS
Park et al. (2019)	In vitro: BV2 mice microglia	10 μM	24 h	100 ng/mL	–	–	↑COX-2, ↑IL-6 mRNA, ↑IL-6,↑IL-1β mRNA, IL-1β, ↑TNF-α mRNA, ↑TNF-α, ↑MIP-1a, ↑MCP-1, ↑IP-10, ↑RANTES, ↑JNK, ↑ERK, ↑AKT, ↑p38 MAPK, ↑NF-κB p65, ↑HO-1	↓COX-2, ↓IL-6, = IL-6 mRNA, ↓IL-1β mRNA, ↓IL-1β, ↓TNF-α, ↓TNF-α mRNA, ↓MIP-1a, ↓MCP-1,= IP-10, = RANTES, ↓JNK, ↓ERK, ↓AKT,= p38 MAPK, = NF-kBp65,= HO-1,	↑NO, ↑iNOS, ↑iNOS mRNA	↓NO, ↓iNOS, ↓iNOS mRNA
Park et al. (2020)	In vitro: BV2 mice microglia	10 μM	24 h	100 ng/mL	–	–	↑IL-1β, ↑TNF-α, ↑IL-6, ↑ERK, ↑JNK, ↑p38 MAPK, ↑AKT, ↑NF-κB	↓IL-1β, ↓TNF-α, ↓IL-6, ↓ERK, ↓JNK, = p38 MAPK, ↓AKT, = NF-κB	↑NO, ↑iNOS mRNA	↓NO, ↓iNOS mRNA
Tynan et al. (2012)	In vitro: BV2 mice microglia	0.1–2.5 μM	4 or 24 h	10 ng/mL	–	–	↑TNF-α	↑TNF-α	↑NO	= NO
		5 μM	4 or 24 h	10 ng/mL	–	–	↑TNF-α	↑TNF-α	↑NO	↓NO
		10–25 μM	4 or 24 h	10 ng/mL	–	–	↑TNF-α	↓TNF-α	↑NO	↓NO
Yang et al. (2014)	In vitro: BV2 mice microglia	0.1 μM	24 h	100 ng/mL	↑Soma size, ↓Process length	–	↑IL-1β, ↓IkBa, ↑NF-κB p65, ↑ERK1/2, ↑p38 MAPK	↓IL-1β, ↑IkBa, = NF-κB p65, ↓ERK1/2, ↓p38 MAPK	–	–
		1 μM	24 h	100 ng/mL	↑Soma size, ↓Process length	–	↑IL-1β, ↓IkBa, ↑NF-κB p65, ↑ERK1/2, ↓p38 MAPK	↓IL-1β, ↑IkBa, ↓NF-κB p65, ↓ERK1/2, ↓p38 MAPK	–	–
		0.1 μM and 0.1 μM acetylsalicylic acid	24 h	100 ng/mL	↑Soma size, ↓Process length	–	↑IL-1β, ↓IkBa, ↑NF-κB p65, ↑ERK1/2, ↓p38 MAPK	↓IL-1β, ↑IkBa, ↓NF-κB p65, ↓ERK1/2, ↓p38 MAPK	–	–
		1 μM and 0.1 μM acetylsalicylic acid	24 h	100 ng/mL	↑Soma size, ↓Process length	–	↑IL-1β	↓IL-1β	–	–
		1 μM	24 h	10 ng/mL	–	–	–	–	↑NO, ↑iNOS mRNA	↓NO, = iNOS mRNA
		3 μM	24 h	10 ng/mL	–	–	↑IKKB, ↑NF-κB p65, ↑p-IkBa, ↓IkBa	↓IKKB, ↓NF-κB p65, ↓p-IkBa, ↑IkBa	↑NO, ↑iNOS mRNA	↓NO, ↓iNOS mRNA
		1 μM	24 h	10 ng/mL	–	–	–	–	↑NO, ↑iNOS mRNA	↓NO, = iNOS mRNA
		3 μM	24 h	10 ng/mL	–	–	↑IKKB, ↑NF-κB p65, ↑p-IkBa, ↓IkBa	↓IKKB, ↓NF-κB p65, ↓p-IkBa, ↑IkBa	↑NO, ↑iNOS mRNA	↓NO, ↓iNOS mRNA
Chung et al. (2010)	Ex vivo: rat Substantia Nigra sections	5/10 mg/kg	8 days	5 mg/3μL	↑OX-42, ↑ED1, ↑amoeboid microglia, ↓Soma size, ↓process length, ↓ramified processes	↓OX-42, ↓ED1, ↓amoeboid microglia, ↑process length, ↑Soma size, ↑ramified processes	–	–	–	–
Rodrigues et al. (2018)	Ex vivo: female Swiss mice hippocampus	10 mg/kg	14 days	Different concentrations over 28 days	↑Iba1	r = Iba1	↑NF-κB p65, ↑IL-1β, = TNF-α	↓NF-κB p65, ↓IL-1β, ↓TNF-α	–	–
Anderson et al. (2016)	Ex vivo: male C57BL/6 mice hippocampus	10 mg/kg	28 days	5 mg/kg	↑Iba1	↓Iba1	–	–	–	–
Paroxetine										
Fujimori et al. (2015)	In vitro: Sprague–Dawley rat primary microglia	1 μM	24 h	10 ng/mL	↑Amoeboid morphology	↓Amoeboid morphology	–	–	–	–
Horikawa et al. (2010)	In vitro: Sprague–Dawley rat primary microglia	0.5 μM	48 h	1 μg/mL	–	–	↑TNF-α	= TNF-α	↑NO	= NO
		0.5 μM	48 h	50 ng/mL	–	–	–	–	↑NO	= NO
		5 μM	48 h	1 μg/mL	–	–	↑TNF-α	= TNF-α	↑NO	↓NO
		5 μM	48 h	50 ng/mL	–	–	–	–	↑NO	= NO
Liu et al. (2014)	In vitro: BV2 mice microglial	5 μM	24 h	0 ng/mL	–	–	–	↓ERK1/2	–	–
		0.1 μM	24 h	100 ng/mL	–	–	↑IL-1β, ↑TNF-α	= IL-1β, = TNF-α	↑NO, ↑iNOS	= NO, = iNOS
		0.2 μM	24 h	100 ng/mL	–	–	↑IL-1β, ↑TNF-α	= IL-1β, = TNF-α	↑NO, ↑iNOS	= NO, = iNOS
		1 μM	24 h	100 ng/mL	–	–	↑IL-1β, ↑TNF-α	↓IL-1β, = TNF-α, ↓JNK1/2	↑NO, ↑iNOS	↓NO, ↓iNOS
		5 μM	24 h	100 ng/mL	–	–	↑IL-1β, ↑TNF-α, ↑IL-1β mRNA, ↑TNF-α mRNA, ↑JNK1/2, ↑NF-κB p65, ↑p38 MAPK	↓IL-1β, ↓TNF-α, ↓IL-1β mRNA, ↓TNF-α mRNA, ↓JNK1/2, = NF-κB p65, = p38 MAPK	↑NO, ↑iNOS	↓NO, ↓iNOS
	In vitro: ICR mice primary microglia	2.5 μM	24 h	100 ng/mL	–	–	↑IL-1β, ↑TNF-α	= IL-1β, = TNF-α	↑NO, ↑iNOS	= NO, = iNOS
		5 μM	24 h	100 ng/mL	–	–	↑IL-1β, ↑TNF-α	= IL-1β, ↓TNF-α	↑NO, ↑iNOS	= NO, = iNOS
		7.5 μM	24 h	100 ng/mL	–	–	↑IL-1β, ↑IL-1β mRNA, ↑TNF-α, ↑TNF-α mRNA	↓IL-1β, ↓TNF-α, ↓IL-1β mRNA, ↓TNF-α mRNA	↑NO, ↑iNOS	↓NO, ↓iNOS
Tynan et al. (2012)	In vitro: BV2 mice microglial	0.1–1 μM	4 or 24 h	10 ng/mL	–	–	↑TNF-α	= TNF-α	↑NO	= NO
		2.5–5 μM	4 or 24 h	10 ng/mL	–	–	↑TNF-α	↑TNF-α	↑NO	= NO
		10–20 μM	4 or 24 h	10 ng/mL	–	–	↑TNF-α	↓TNF-α	↑NO	↓NO
Citalopram										
Dhami et al. (2013)	In vitro: Sprague–Dawley rat primary microglia	10 μM	24 h	1 μg/mL	–	–	↑IL-1β, ↑TNF-α	↓IL-1β, ↓TNF-α	↑NO	↓NO
Tynan et al. (2012)	In vitro: BV2 mice microglial	0.1–5 μM	4 or 24 h	10 ng/mL	–	–	↑TNF-α	= TNF-α	↑NO	= NO
		10–35 μM	4 or 24 h	10 ng/mL	–	–	↑TNF-α	↓TNF-α	↑NO	= NO
**Sertraline**										
Horikawa et al. (2010)	In vitro: Sprague–Dawley rat primary microglia	0.5 μM	48 h	1 μg/mL	–	–	↑TNF-α	= TNF-α	↑NO	= NO
		0.5 μM	48 h	50 ng/mL	–	–	–	–	↑NO	= NO
		5 μM	48 h	1 μg/mL	–	–	↑TNF-α	= TNF-α	↑NO	↓NO
		5 μM	48 h	50 ng/mL	–	–	–	–	↑NO	= NO
Tynan et al. (2012)	In vitro: BV2 mice microglia	0.1–1 μM	4 or 24 h	10 ng/ml	–	–	↑TNF-α	= TNF-α	↑NO	= NO
		2.5 μM	4 or 24 h	10 ng/mL	–	–	↑TNF-α	↑TNF-α	↑NO	= NO
		5 μM	4 or 24 h	10 ng/mL	–	–	↑TNF-α	= TNF-α	↑NO	= NO
		10–20 μM	4 or 24 h	10 ng/mL	–	–	↑TNF-α	↓TNF-α	↑NO	↓NO
Norfluoxetine										
Dhami et al. (2019)	In vitro: Sprague–Dawley rat; primary microglia	10 μM	24 h	1 μg/mL	–	–	↑TNF-α	↓TNF-α	↑NO	↓NO
Kim et al. (2018)	In vitro: Sprague–Dawley rat; primary microglia	10 μM	12 h	100 μg/mL	–	–	–	–	↑NO	= NO
		50 μM	12 h	100 μg/mL	–	–	–	–	↑NO	↓NO
		100 μM	12 h	100 μg/mL	–	–	–	–	↑NO	↓NO
Fluvoxamine										
Tynan et al. (2012)	In vitro: BV2 mice microglia	0.1–5 μM	4 or 24 h	10 ng/mL	–	–	↑TNF-α	↑TNF-α	↑NO	= NO
		10–25 μM	4 or 24 h	10 ng/mL	–	–	↑TNF-α	↓TNF-α	↑NO	= NO
SNRI antidepressants										
Venlafaxine										
Tynan et al. (2012)	In vitro: BV2 mice microglia	0.1 μM	4 or 24 h	10 ng/mL	–	–	↑TNF-α	= TNF-α	↑NO	= NO
		1 μM	4 or 24 h	10 ng/mL	–	–	↑TNF-α	↑TNF-α	↑NO	= NO
		2.5 μM	4 or 24 h	10 ng/mL	–	–	↑TNF-α	= TNF-α	↑NO	= NO
		10 μM	4 or 24 h	10 ng/mL	–	–	↑TNF-α	↓TNF-α	↑NO	= NO
		15–35 μM	4 or 24 h	10 ng/mL	–	–	↑TNF-α	= TNF-α	↑NO	= NO
Dubovický et al. (2014)	In vitro: BV2 mice microglia	25 μmol/L	24 h	1 μg/mL	↑Latex bead phagocytosis, ↑Cells	r = Latex bead phagocytosis, = Cells	–	–	↑NO	= NO
		50 mol/L	24 h	1 μg/mL	↑Latex bead phagocytosis, ↑Cells	r = Latex bead phagocytosis, = Cells	–	–	↑NO	= NO
		100 μmol/L	24 h	1 μg/mL	↑Latex bead phagocytosis, ↑Cells	↓Latex bead phagocytosis, = Cells	–	–	↑NO	↓NO
		25 μmol/L	24 h	10 μg/mL	↓Mitochondrial V_m_, ↓Lysosomal stability	r = Mitochondrial V_m_, ↑Lysosomal stability	–	–	–	–
		50 μmol/L	24 h	10 μg/mL	↓Mitochondrial V_m_, ↓Lysosomal stability	r = Mitochondrial V_m_, ↑Lysosomal stability	–	–	–	–
		100 μmol/L	24 h	10 μg/mL	↓Mitochondrial V_m_, ↓Lysosomal stability	↑Mitochondrial V_m_, ↑Lysosomal stability	–	–	–	–
Amitriptyline										
Obuchowicz et al. (2006)	In vitro: Wistar Rat primary microglia	1 μM	24 h	2 μg/mL	–	–	↑IL-1β	↓IL-1β	-	-
		10 μM	24 h	2 μg/mL	–	–	↑IL-1β	↓IL-1β	-	-
		50 μM	24 h	2 μg/mL	–	–	↑IL-1β	↓IL-1β	-	-
Park et al. (2019)	In vitro: BV2 mice microglia	10 μM	24 h	100 ng/mL	–	–	↑COX-2, ↑IL-6 mRNA, ↑IL-6, ↑IL-1β mRNA, ↑IL-1β, ↑TNF-α mRNA, ↑TNF-α, ↑MIP-1a, ↑MCP-1, ↑IP-10, ↑RANTES, ↑JNK, ↑ERK, ↑AKT, ↑p38 MAPK, ↑NF-κB p65, ↑HO-1	= COX-2, ↓IL-6 mRNA, ↓IL-6, ↓IL-1β mRNA, ↓IL-1β, ↓TNF-α mRNA, ↓TNF-α, ↓MIP-1a, ↓MCP-1, ↓IP-10, = RANTES, ↓JNK, ↓ERK, ↓AKT, = p38 MAPK, = NF-κB p65, = HO-1	↑NO, ↑iNOS, ↑iNOS mRNA	= NO, ↓iNOS, = iNOS mRNA
Imipramine										
Dhami et al. (2013)	In vitro: Sprague–Dawley rat primary microglia	10 μM	24 h	1 μg/mL	–	–	↑IL-1β, ↑TNF-α	↓TNF-α, ↓IL-1β	↑NO	↓NO
Clomipramine										
Dhami et al. (2013)	In vitro: Sprague–Dawley rat primary microglia	10 μM	24 h	1 μg/mL	–	–	↑TNF-α,↑IL-1β	= TNF-α, ↓IL-1β	↑NO	↓NO
Gong et al. (2019)	Ex vivo: male C57BL/6 mice hippocampus	10 μM	2, 6 and 12 h	1 ng/mL	↑CD11b, ↑IbA1	= CD11b, ↓IbA1	–	–	–	–
	In vitro: BV2 mice microglia	10 μM	2, 6 and 12 h	1 ng/mL	–	–	↑IL-1β mRNA, ↑IL-6 mRNA, ↑IL-18 mRNA, ↑TNF-α mRNA, ↑IL-1β, ↑IL-6, ↑TNF-α, ↑NLRP3,	↓IL-1β mRNA, ↓IL-6 mRNA, = IL-18 mRNA, = TNF-α mRNA, = IL-1β, ↓IL-6, ↓TNF-α, ↓NLRP3,	–	–
	In vitro: C57BL/6 mice primary microglia	10 μM	2, 6 and 12 h	1 ng/mL	–	–	↑IL-1β mRNA, ↑IL-6 mRNA, ↑IL-18 mRNA, ↑TNF-α mRNA, ↑IL-1β, ↑IL-6, ↑IL-18, ↑TNF-α, ↑NLRP3, ↑ASC, ↑Caspase-1	↓IL-1β mRNA, ↓IL-6 mRNA, ↓IL-18 mRNA, = TNF-α mRNA, = IL-1β, = IL-18, ↓IL-6, = TNF-α, = NLRP3, = ASC, = Caspase-1	–	–
Monoamine oxidase inhibitors										
Phenelzine										
Chung et al. (2012)	In vitro: BV2 mice microglia	1 μM	24 h	0.2 μg/mL	–	–	↑IL-6, ↑TNF-α	= IL-6, = TNF-α	↑NO, ↑iNOS mRNA, ↑iNOS	↑NO, = iNOS mRNA, ↑iNOS
		10 μM	24 h	0.2 μg/mL	–	–	↑IL-6, ↑TNF-α	↑IL-6, = TNF-α	↑NO, ↑iNOS mRNA, ↑iNOS	↑NO, = iNOS mRNA, ↑iNOS
		50 μM	24 h	0.2 μg/mL	–	–	↑IL-6, ↑TNF-α, ↑NF-κB p65, ↑p-IkBa	↑IL-6, ↑TNF-α, ↑NF-κB p65, ↑p-IkBa	↑NO, ↑iNOS mRNA, ↑iNOS	↑NO, ↑iNOS mRNA, ↑iNOS
	In vitro: C57BL/6 mice; primary microglia	1 μM	24 h	0.2 μg/mL	–	–	↑IL-6, ↑TNF-α, ↑IL-6 mRNA, ↑TNF-α mRNA	= IL-6, = TNF-α, = IL-6 mRNA, = TNF-α mRNA	↑NO	↑NO
		10 μM	24 h	0.2 μg/mL	–	–	↑IL-6, ↑TNF-α, ↑IL-6 mRNA, ↑TNF-α mRNA	↑IL-6, = TNF-α, = IL-6 mRNA, = TNF-α mRNA	↑NO	↑NO
		50 μM	24 h	0.2 μg/mL	–	–	↑IL-6, ↑TNF-α, ↑IL-6 mRNA, ↑TNF-α mRNA	↑IL-6, ↑TNF-α, = IL-6 mRNA, ↑TNF-α mRNA	↑NO	↑NO
Dhami et al. (2013)	In vitro: Sprague–Dawley rat primary microglia	10 μM	24 h	1 μg/mL	–	–	↑IL-1β, ↑TNF-α	= TNF-α, ↓IL-1β	↑NO	↓NO
Tranylcypromine										
Dhami et al. (2013)	In vitro: Sprague–Dawley rat primary microglia	10 μM	24 h	1 μg/mL	–	–	↑IL-1β, ↑TNF-α	↓TNF-α, ↓IL-1β	↑NO	↓NO
Park et al. (2020)	Ex vivo: male C57BL6/N mice cortex	3 mg/kg	24 h	10 mg/kg	↑IbA1	↓IbA1	–	–	–	–
	Ex vivo: male C57BL6/N mice CA1	3 mg/kg	24 h	10 mg/kg	↑IbA1	↓IbA1	–	–	–	–
	Ex vivo: male C57BL6/N mice dentate gyrus	3 mg/kg	24 h	10 mg/kg	↑IbA1	↓IbA1	–	–	–	–
	In vitro: BV2 mice microglia	5 μM	24 h	1 μg/mL	–	–	↑IL-6 mRNA, ↑IL-1β mRNA,↑IL-6, ↑TNF-α,↑ERK,↑STAT3, ↑NF-κB	↓IL-6 mRNA, ↓IL-1β mRNA, ↓IL-6, = TNF-α, ↓ERK, ↓STAT3, ↓NF-κB	↑iNOS	= iNOS
Others										
Ketamine										
Lu et al. (2019)	In vitro: BV2 mice Microglia	1 μg/mL	24 h	1 μg/mL			COX II, iNOS, IL-1a, and TNF-a COX II, iNOS, IL-1a, and TNF-a ↑ COX II mRNA, ↑ IL-1a mRNA and ↑ TNF-a mRNA	= COX II mRNA, = IL-1a mRNA, and = TNF-a mRNA	↑ iNOS	= iNOS
		10 μg/mL	24 h	1 μg/mL			↑ COX II mRNA, ↑ IL-1a mRNA and ↑ TNF-a mRNA	↓ COX II mRNA, ↓ IL-1a mRNA and ↓ TNF-a mRNA	↑ iNOS	↓ iNOS
		20 μg/mL	24 h	1 μg/mL			↑ COX II mRNA, ↑ IL-1a mRNA and ↑ TNF-a mRNA	↑ COX II mRNA, ↓ IL-1a mRNA and ↓ TNF-a mRNA	↑ iNOS	↓ iNOS

SSRI: selective serotonin re-uptake inhibitor; SNRI: serotonin and norepinephrine re-uptake inhibitors; LPS: lipopolysaccharide; INF: interferon; IL: interleukin; TNF: tumour necrosis factor; NO: nitric oxide; NOS: nitric oxide synthase; mRNA: messenger RNA; NF-κB: nuclear factor kappa light-chain enhancer of activated B cell; ↑: significant increase; ↓: significant decrease; =: no significant change.

Five studies showed the ability of fluoxetine to prevent microglial cellular changes induced by treatment with LPS *in vitro* and *ex vivo* (Anderson et al., 2016; Chung et al., 2010; Liu et al., 2011; Rodrigues et al., 2018; Zhang et al., 2012). In particular, Zhang et al. (2012) showed that treatment with fluoxetine prevented LPS-induced increase in ionised calcium-binding adaptor molecule 1 (IbA1) expression and morphological changes, such as larger cell bodies, thicker processes and irregular shapes, in primary rat microglial cells. Moreover, Liu et al. (2011) showed that treatment with fluoxetine, followed by the LPS challenge, reduced CD11b expression in mice primary microglial cells. Similarly to the in vitro studies, two ex vivo studies showed that treatment with fluoxetine prevented LPS-induced increase in OX-42 and ED1 microglia marker expression in rats substantia nigra (Chung et al., 2010), and increase in IbA1 expression in mice hippocampi (Anderson et al., 2016). However, in another ex vivo study using the same experimental model, Rodrigues et al. did not find any effect of fluoxetine on LPS-induced increase in IbA1 in mice hippocampi (Rodrigues et al., 2018). The hippocampus is an area of the brain responsible for neurogenesis, the process through which new neurons are generated in the brain, and it is known to regulate both memory and emotion, all of which are dysregulated patients with depression (Boldrini et al., 2014, 2012; Sheline et al., 1996).

Three of the studies previously mentioned (Liu et al., 2011; Rodrigues et al., 2018; Zhang et al., 2012) and seven additional studies (Dhami et al., 2013; Du et al., 2014, 2016; Park et al., 2019, 2020; Tynan et al., 2012; Yang et al., 2014) also showed the ability of fluoxetine to prevent the production of microglial immune markers induced by LPS in vitro and ex vivo. In fact, fluoxetine prevented LPS-induced production of IL-1β, IL-6 and TNF-α, as well as other biomarkers of inflammation in primary rat and mice microglia cultures (Dhami et al., 2013; Du et al., 2014, 2016; Liu et al., 2011; Zhang et al., 2012) and mice BV2 microglia cultures (Liu et al., 2011; Park et al., 2019; Tynan et al., 2012; Yang et al., 2014), and in mice hippocampi (Rodrigues et al., 2018). In addition, fluoxetine prevented an LPS-induced increase in *IL-1β*, *TNF-α* and *IL-6* gene expression (Liu et al., 2011; Park et al., 2019). Importantly, most of these studies showed that the anti-inflammatory property of fluoxetine is mediated by inhibition of nuclear factor kappa-light-chain-enhancer of activated B cells (NF-κB), mitogen-activated protein kinase (MAPK) and extracellular signal-regulated kinases (ERK1/2) pathways (Du et al., 2014; Liu et al., 2011; Park et al., 2019; Yang et al., 2014; Zhang et al., 2012).

Some of the above-mentioned studies also showed the ability of fluoxetine to prevent the production of microglial oxidative stress markers induced by LPS in vitro (Dhami et al., 2013; Du et al., 2014; Liu et al., 2014; Park et al., 2019, 2020; Tynan et al., 2012; Yang et al., 2014; Zhang et al., 2012). In particular, fluoxetine prevented LPS-induced production of nitric oxide (NO) in primary rat and mice microglia cells (Dhami et al., 2013; Du et al., 2014; Liu et al., 2011; Zhang et al., 2012) and in mice BV2 microglia cells (Liu et al., 2011; Park et al., 2019, 2020; Tynan et al., 2012). In addition, in the same studies, fluoxetine prevented LPS-induced increase in nitric oxide synthases (*iNOS*) gene expression, a key enzyme for NO production (Liu et al., 2011; Park et al., 2019, 2020; Zhang et al., 2012).

##### Paroxetine

Four in vitro studies investigated the effect of paroxetine on LPS-induced microglial activation (Table 1). Overall, the studies showed that paroxetine prevented microglial morphological changes, as well as immune activation and oxidative stress production upon in vitro exposure to LPS.

Only one study investigated the ability of paroxetine to prevent cellular changes induced by treatment with LPS (Fujimori et al., 2015). In their study, Fujimori et al. (2015) found an increase in amoeboid morphology in rat primary microglial cultures, which was reduced by paroxetine.

Two of the studies previously mentioned (Liu et al., 2011; Tynan et al., 2012) and another study (Horikawa et al., 2010) have also investigated the ability of paroxetine to prevent the production of microglial immune markers. In particular, Horikawa et al. (2010) did not find any effect of paroxetine on LPS-induced production of TNF-α in rat primary microglia, with LPS used at a concentration of 1 μg/mL, while Liu et al. (2014), using a different concentration of LPS (100 ng/mL), found that paroxetine prevented LPS-induced *IL-1β* and *TNF-α* genes and protein expression in both mice primary microglia and mice BV2 microglia cells. Moreover, in mice BV2 cells, paroxetine prevented LPS-induced c-Jun N-terminal kinases (JNK1/2) activation, but did not affect MAPK p38 and NF-κB p65 activation (Liu et al., 2014). In addition, Tynan et al. (2012) showed that 2.5 and 5 μM paroxetine enhanced the increase in TNF-α production upon LPS treatment, whereas paroxetine above 10 μM prevented LPS-induced TNF-α production, in mice BV2 microglial cells.

All of the above studies also showed the ability of paroxetine to prevent the production of microglial oxidative stress markers (Horikawa et al., 2010; Liu et al., 2011; Tynan et al., 2012). In particular, Horikawa et al. (2010) showed that paroxetine prevented LPS-induced release of NO in rat primary microglial culture, with LPS used at a concentration of 1 μg/mL. However, paroxetine did not affect NO release when a lower concentration of LPS (50 ng/mL) was used (Horikawa et al., 2010). On the contrary, Tynan et al. (2012) used a much lower concentration of LPS (10 ng/mL) and showed that 10–20 μM paroxetine prevented LPS-induced NO production in mice BV2 microglial cultures. Moreover, Liu et al. (2014) showed that 1 and 7.5 μM paroxetine prevented LPS-induced increase in *iNOS* gene expression and NO production in mice BV2 and mice primary microglial cultures, respectively.

##### Citalopram

Two previously mentioned in vitro studies also investigated the effects of citalopram on LPS-induced microglial activation (Table 1; Dhami et al., 2013; Tynan et al., 2012). They showed that citalopram prevented immune activation, but did not have any effect on oxidative stress production upon in vitro exposure to LPS.

Dhami et al. (2013) showed that citalopram prevented LPS-induced production of IL-1β, TNF-α and NO in rat primary microglia. In contrast, Tynan et al. (2012) showed that only concentrations of citalopram higher than 10 μM prevented LPS-induced production of TNF-α, whereas treatment with citalopram did not have any effect on LPS-induced NO production in mice BV2 microglial cells.

##### Sertraline

Two previously mentioned in vitro studies also investigated the effects of sertraline on LPS-induced microglial activation (Table 1; Horikawa et al., 2010; Tynan et al., 2012). They showed that sertraline prevented immune activation and oxidative stress production upon in vitro exposure to LPS, although this outcome was observed only upon treatment with specific concentrations of sertraline.

In particular, Horikawa et al. (2010) showed that sertraline prevented LPS-induced TNF-α production in rat primary microglial cells, as well as of NO production, but in this case only with the concentration of sertraline and LPS, respectively, equal to 5 μM and 1 μg/mL. In contrast, Tynan et al. (2012) showed that only concentrations of sertraline higher than 10 μM prevented LPS-induced TNF-α and NO production in mice BV2 microglial cells.

##### Norfluoxetine

Two additional in vitro studies investigated the effect of norfluoxetine on LPS-induced microglial activation (Table 1; Dhami et al., 2019; Kim et al., 2018). Overall, these studies showed that norfluoxetine prevented immune activation and oxidative stress production upon *in vitro* exposure to LPS.

In particular, Dhami et al. (2019) showed that norfluoxetine prevented LPS-induced increase in TNF-α and NO production in rat primary microglial cultures. However, Kim et al. (2018) showed that only concentrations of norfluoxetine higher than 50 μM prevented LPS-induced NO production in rat primary microglial cultures.

##### Fluvoxamine

To our knowledge, only one in vitro study examined the effect of fluvoxamine on LPS-induced microglial activation (Table 1). In particular, Tynan et al. (2012) found that only concentrations of fluvoxamine higher than 10 μM prevented LPS-induced production of TNF-α, but not NO, in mice BV2 microglial cultures.

#### Serotonin–norepinephrine reuptake inhibitors

##### Venlafaxine

Only one study previously mentioned (Tynan et al., 2012) and one additional study (Dubovický et al., 2014), both in vitro, have investigated the effect of venlafaxine on LPS-induced microglial activation (Table 1). Overall, these studies showed that only at specific concentrations, venlafaxine prevents immune activation, but did not have any effect on oxidative stress production upon in vitro exposure to LPS.

In particular, in the first study, Dubovický et al. (2014) showed that only concentrations of venlafaxine equal to 100 µM prevented LPS-induced cell phagocytosis and exerted also a protective effect on mitochondrial membrane potential in mice BV2 microglial culture. In the second study, Tynan et al. (2012) investigated the effects of venlafaxine on microglial immune changes induced by LPS, and showed that only concentrations of venlafaxine equal to 10 μM prevented LPS-induced reduction in TNF-α in mice BV2 microglial culture.

Finally, both studies investigated the effect of different venlafaxine concentrations on microglial oxidative stress products after exposure to LPS (Dubovický et al., 2014; Tynan et al., 2012). Dubovický et al. (2014) showed that concentrations of venlafaxine equal to 100 µmol/L partially prevented LPS-induced NO production, whereas the Tynan et al (2012) study showed that irrespective of the range of concentrations used (0.1, 1, 2.5, 10 and from 15 to 35 μM), venlafaxine did not prevent LPS-induced NO production.

#### Tricyclic antidepressants (TCAs)

##### Amitriptyline

One previously mentioned study (Park et al., 2019) and an additional study (Obuchowicz et al., 2006), both in vitro, investigated the effect of amitriptyline on LPS-induced microglial activation (Table 1). Both studies showed that amitriptyline prevented immune activation and oxidative stress production upon in vitro exposure to LPS.

Obuchowicz et al. (2006) showed that only concentrations of amitriptyline higher than 1 μM prevented the production of LPS-induced increase in IL-1β in rat primary microglial cells. Similarly, Park et al. (2019) showed that 10 μM amitriptyline prevented LPS-induced IL-1β, TNF-α, IL-6 and iNOS production, as well as *IL-1β*, *TNF-α* and *IL-6* gene expression, in mice BV2 microglial cells. However, there was no effect of amitriptyline on the LPS-induced increase in NF-κB pathway activation.

##### Imipramine

Only one in vitro study, previously mentioned (Dhami et al., 2013), investigated the effect of imipramine on LPS-induced microglial activation (Table 1). In their study, Dhami et al. (2013) showed that imipramine is able to restore LPS-induced increased production of TNF-α and IL-1β, and NO in rat primary microglial cultures.

##### Clomipramine

Two additional studies, conducted both in vitro and ex vivo experiments, investigated the effects of clomipramine on LPS-induced microglial activation (Table 1; Dhami et al., 2013; Gong et al., 2019). Overall, both studies showed that clomipramine prevented microglial cellular changes, as well as immune activation and oxidative stress production upon in vitro and ex vivo exposure to LPS.

Gong et al. (2019) showed that clomipramine prevented the expression of the microglial marker IbA1 in the hippocampus of LPS-treated mice. Moreover, in the same study, in vitro treatment with clomipramine prevented LPS-induced IL-1β, IL-6 and TNF-α production and gene expression in mice primary microglial cells and in mice BV2 microglial cells, whereas in vitro treatment with clomipramine also prevented LPS-induced increase in NLR family pyrin domain containing 3 (*NLRP3*) gene expression in mice BV2 cells (Gong et al., 2019). However, Dhami et al. (2013) showed that only concentrations of clomipramine equal to 10 μM prevented LPS-induced IL-1β and NO, but not TNF-α, production.

#### Monoamine oxidase inhibitors

##### Phenelzine

Two previously mentioned in vitro studies investigated the effect of phenelzine on LPS-induced microglial activation (Chung et al., 2012; Dhami et al., 2013; Table 1). Overall, one study found the ability of phenelzine to prevent microglial production of immune and oxidative stress outcomes upon treatment with LPS, whereas the second study showed the opposite. Interestingly, such contrasting findings might be due to the use of different concentrations of LPS (respectively, 1 and 0.2 μg/ml).

In particular, Dhami et al. (2013) showed that 10 μM phenelzine is able to prevent LPS-induced increase in IL-1β protein levels in rat primary microglia, but there was no effect on TNF-α protein levels. However, Chung et al. (2012) showed that 10 and 50 μM phenelzine further increased LPS-induced production of, respectively, IL-6 and TNF-α, in both mice BV2 and mice primary microglial cells. In addition, phenelzine 50 μM increased LPS-induced NF-κB nuclear accumulation in mice BV2 microglial cells (Chung et al., 2012).

Dhami et al. (2013) also showed that phenelzine prevented LPS-induced increase in NO production. However, Chung et al. showed that phenelzine further increased LPS-induced NO production in both mice BV2 and mice primary microglial cells, and iNOS and *iNOS* gene expression in mice primary microglial cell.

##### Tranylcypromine

Two previously mentioned in vitro and ex vivo studies investigated the effect of tranylcypromine on LPS-induced microglial activation (Table 1). Overall, the studies showed that tranylcypromine prevented microglial cellular changes as well as immune activation and oxidative stress production upon in vitro and ex vivo exposure to LPS.

In an ex vivo study, Park et al. (2020) showed that tranylcypromine partially prevented the expression of the microglial marker IbA1 in the cortex, hippocampus and dentate gyrus of LPS-treated mice. In the same study, in vitro treatment with tranylcypromine prevented LPS-induced production of IL-1β, IL-6, IL-4 and ERK, signal transducer and activator of transcription 3 (STAT3) and NF-κB protein expression in mice BV2 microglial cells (Park et al., 2020). Similarly, in the second in vitro study, Dhami et al. (2013) showed that treatment with tranylcypromine prevented LPS-induced production of TNF-α, IL-1β, in rat primary microglial cells. Finally, Dhami et al. (2013) showed that tranylcypromine prevented LPS-induced increase in NO production in rat primary microglial cells.

#### Ketamine

Only one in vitro study showed that treatment with ketamine prevented gene and protein expression of proinflammatory cytokines, oxidative stress molecules and related enzymes in a concentration-dependent manner in LPS-induced BV2 microglial cells (Lu et al., 2020). In particular, 10 and 20 μg/μL of ketamine were able to reduce the levels of COX II, iNOS, IL-1a, and TNF-a mRNA expression increased by the LPS challenge.

### Cytokines-induced models of neuroinflammation

This section summarises the findings of five studies, which reported the effect of SSRIs, tricyclic, norepinephrine reuptake inhibitors (NRIs) and atypical antidepressants in preventing microglial activation induced by cytokines treatment in in vitro and ex vivo models of neuroinflammation. As previously mentioned, microglial activation has been identified by cellular changes and/or by the presence of inflammatory and/or oxidative stress products typical of an activated status.

#### Selective serotonin re-uptake Inhibitors

##### Fluoxetine

Only one in vitro study, investigated the effect of fluoxetine on IL-4-induced microglial activation (Su et al., 2015; Table 2). The study showed that treatment with fluoxetine increased IL-4-induced expression of the microglial M2 surface marker CD206 and *IL-10* gene expression both in mice BV2 cells and rat primary microglial cells (Su et al., 2015).

**Table 2.** Studies examining the effects of antidepressants in preventing microglial activation induced by cytokines in in vitro and ex vivo models of neuroinflammation.

Article	Investigated tissue	Drug concentration	Duration	Cytokines concentration	Microglial activation outcomes					
					Cellular modulation		Oxidative stress		Inflammation	
					LPS (vs control)	LPS + Antidepressant (vs LPS)	LPS (vs control)	LPS + Antidepressant (vs LPS)	LPS (vs control)	LPS + Antidepressant (vs LPS)
SSRI antidepressants										
Fluoxetine (FLX)										
Su et al. (2015)	In vitro: BV2 mice microglia	20 µM	24 h	IL-4 (10 ng/mL)	↑CD206	↑CD206			↑IL-10 mRNA, ↑IL-10	= IL-10 mRNA, ↑IL-10
		60 µM	24 h	IL-4 (10 ng/mL)	↑CD206	↑CD206			↑IL-10 mRNA, ↑IL-10	↑IL-10 mRNA, ↑IL-10
		20 µM	24 h	LPS (200 ng/mL) + IFN-γ (20 ng/mL)	↑CD68	↓CD68	↑iNOS	↓iNOS	↑IL-6 mRNA, ↑IL-1β mRNA, ↑TNFα mRNA, ↑IL-1β, ↑TNFα	↓IL-6 mRNA, ↓IL-1β mRNA, ↓TNFα mRNA, ↓IL-1β
		60 µM	24 h	LPS (200 ng/mL) + IFN-γ (20 ng/mL)	↑CD68	↓CD68	↑iNOS	↓iNOS	↑IL-6 mRNA, ↑IL-1β mRNA, ↑TNFα mRNA, ↑IL-1β, ↑TNFα	↓IL-6 mRNA, ↓IL-1β mRNA, ↓TNFα mRNA, ↓IL-1β
	In vitro: Sprague–Dawley rat; primary microglia	20 µM	24 h	IL-4 (10 ng/mL)	↑CD206	↑CD206			↑IL-10 mRNA, ↑IL-10	↑IL-10 mRNA, ↑IL-10
		60 µM	24 h	IL-4 (10 ng/mL)	↑CD206	↑CD206			↑IL-10 mRNA, ↑IL-10	↑IL-10 mRNA, ↑IL-10
		20 µM	24 h	LPS (200 ng/mL) + IFN-γ (20 ng/mL)	↑CD68	↓CD68	↑iNOS	↓iNOS	↑IL-6 mRNA, ↑IL-1β mRNA, ↑TNFα mRNA, ↑IL-1β, = TNFα	↓IL-6 mRNA, = IL-1β mRNA, = TNFα mRNA, ↓IL-1β, = TNFα
		60 µM	24 h	LPS (200 ng/mL) + IFN-γ (20 ng/mL)	↑CD68	↓CD68	↑iNOS	↓iNOS	↑IL-6 mRNA, ↑IL-1β mRNA, ↑TNFα mRNA, ↑IL-1β, = TNFα	↓IL-6 mRNA, ↓IL-1β mRNA, ↓TNFα mRNA, ↓IL-1β, = TNFα
Paroxetine (PRX)										
Horikawa et al. (2010)	In vitro: Murine 6-3 microglia	0.5 µM	48 h	IFN-γ (50 U/mL)	= MTT absorbance	= MTT absorbance	↑NO	= NO	↑TNFα	= TNFα
		5 µM	48 h	IFN-γ (50 U/mL)	= MTT absorbance, ↑[Ca^2+^]i	= MTT absorbance, ↓[Ca^2+^]i	↑NO	↓NO	↑TNFα, = IL-4	↓TNFα, = IL-4
Citalopram (CIT)										
Su et al. (2015)	In vitro: BV2 mice microglia	20µM	24 h	IL-4 (10 ng/mL)	↑CD206	= CD206			↑ IL-10 mRNA, ↑IL-10	= IL-10 mRNA, = IL-10
		60 µM	24 h	IL-4 (10 ng/mL)	↑CD206	↑CD206			↑IL-10 mRNA, ↑IL-10	= IL-10 mRNA, ↑IL-10
		20 µM	24 h	LPS (200 ng/mL) + IFN-γ (20 ng/mL)	↑CD68	↓CD68	↑iNOS	= iNOS	↑IL-6 mRNA, ↑IL-1β mRNA, ↑TNFα mRNA, ↑IL-1β, ↑TNFα	= IL-6 mRNA, = IL-1β mRNA, = TNFα mRNA, = IL-1β, = TNFα
		60 µM	24 h	LPS (200 ng/mL) + IFN-γ (20 ng/mL)	↑CD68	↓CD68	↑iNOS	↓iNOS	↑IL-6 mRNA, ↑IL-1β mRNA, ↑TNFα mRNA, ↑IL-1β, ↑TNFα	↓IL-6 mRNA, = IL-1β mRNA, ↓TNFα mRNA, ↓IL-1β, = TNFα
	In vitro: Sprague–Dawley rat; primary microglia	20 µM	24 h	IL-4 (10 ng/mL)	↑CD206	= CD206			↑IL-10 mRNA, ↑IL-10	= IL-10 mRNA, ↓IL-10
		60 µM	24 h	IL-4 (10 ng/mL)	↑CD206	↑CD206			↑IL-10 mRNA, ↑IL-10	↑IL-10 mRNA, ↑IL-10
		20 µM	24 h	LPS (200 ng/mL) + IFN-γ (20 ng/mL)	↑CD68	↓CD68	↑iNOS	↓iNOS	↑IL-6 mRNA, ↑IL-1β mRNA, ↑TNFα mRNA, ↑IL-1β, = TNFα	↓IL-6 mRNA, = IL-1β mRNA, = TNFα mRNA, ↓IL-1β, = TNFα
		60 µM	24 h	LPS (200 ng/mL) + IFN-γ (20 ng/mL)	↑CD68	↓CD68	↑iNOS	↓iNOS	↑IL-6 mRNA, ↑IL-1β mRNA, ↑TNFα mRNA, ↑IL-1β, = TNFα	↓IL-6 mRNA, ↓IL-1β mRNA, ↓TNFα mRNA, ↓IL-1β, = TNFα
Escitalopram (ECIT)										
Wang et al. (2018a)	In vivo: male C57BL/6 J mice dorsal raphe nucleus	10 mg/kg	21 days	IFN-α (1.5×10^7^ U/kg)	↑Iba1	↓Iba1	–	–	–	–
Sertraline (SERT)										
Horikawa et al. (2010)	In vitro: Murine 6-3 microglia	0.5 µM	48 h	IFN-γ (50 U/mL)	= MTT absorbance	= MTT absorbance	↑NO	R = NO	↑TNFα	R = TNFα
		5 µM	48 h	IFN-γ (50 U/mL)	= MTT absorbance, ↑[Ca^2+^]i	= MTT absorbance, ↓[Ca^2+^]i	↑NO	↓NO	↑TNFα, = IL-4	↓TNFα, = IL-4
Lu et al. (2019)	In Vitro: BV2 mice microglia	0.5 µM	24 h	TNF-α (10 ng/mL)	↑Iba1	↓Iba1	↑iNOS	↓iNOS	↑TNFα	= TNFα
		1 µM	24 h	TNF-α (10 ng/mL)	↑Iba1	↓Iba1	↑iNOS	↓iNOS	↑TNFα, ↓IkBa, ↑NF-κB p65	↓TNFα, ↓NF-κB p65
Fluvoxamine (FLV)										
Hashioka et al. (2007)	In vitro: Murine 6-3 microglia	10µM	24 h	IFN-γ (100 U/mL)	–	–	↑NO	↓NO	↑IL-6	↓IL-6
		50 µM	24 h	IFN-γ (100 U/mL)	–	–	↑NO	↓NO	↑IL-6	↓IL-6
		100 µM	24 h	IFN-γ (100 U/mL)	–	–	↑NO	↓NO	↑IL-6	↓IL-6
		50 µM + SQ 22,536 (10 µM)	24 h	IFN-γ (100 U/mL)	–	–	↑NO	= NO	↑IL-6	= IL-6
		50 µM + Rp-3′,5′- cAMPS (10 µM)	24 h	IFN-γ (100 U/mL)	–	–	↑NO	= NO	↑IL-6	= IL-6
Tricylic antidepressants										
Imipramine (IMI)										
Hashioka et al. (2007)	In vitro: Murine 6-3 microglia	10 µM	24 h	IFN-γ (100 U/mL)	–	–	↑NO	= NO	↑IL-6	↓IL-6
		50 µM	24 h	IFN-γ (100 U/mL)	–	–	↑NO	↓NO	↑IL-6	↓IL-6
		100 µM	24 h	IFN-γ (100 U/mL)	–	–	↑NO	↓NO	↑IL-6	↓IL-6
		50 µM + SQ 22,536 (10 µM)	24 h	IFN-γ (100 U/mL)	–	–	↑NO	= NO	↑IL-6	= IL-6
		50 µM + Rp-3′,5′- cAMPS (10 µM)	24 h	IFN-γ (100 U/mL)	–	–	↑NO	= NO	↑IL-6	= IL-6
NRI antidepressants										
Reboxetine (RBX)										
Hashioka et al. (2007)	In vitro: Murine 6-3 microglia	10 µM	24 h	IFN-γ (100 U/mL)	–	–	↑NO	↓NO	↑IL-6	= IL-6
		50 µM	24 h	IFN-γ (100 U/mL)	–	–	↑NO	↓NO	↑IL-6	↓IL-6
		100 µM	24 h	IFN-γ (100 U/mL)	–	–	↑NO	↓NO	↑IL-6	↓IL-6
		50 µM + SQ 22,536 (10 µM)	24 h	IFN-γ (100 U/mL)	–	–	↑NO	= NO	↑IL-6	= IL-6
		50 µM + Rp-3′,5′- cAMPS (10 µM)	24 h	IFN-γ (100 U/mL)	–	–	↑NO	= NO	↑IL-6	= IL-6
Atypical antidepressants										
Bupropion (BPN)										
Horikawa et al. (2010)	In vitro: Murine 6-3 microglia	1 µM	48 h	IFN-γ (50 U/mL)	= MTT absorbance	= MTT absorbance	↑NO	= NO	–	–
		10 µM	48 h	IFN-γ (50 U/mL)	= MTT absorbance, ↑[Ca^2+^]	= MTT absorbance, =[Ca^2+^]i	↑NO	= NO	–	–
Agomelatine (AGM)										
Horikawa et al. (2010)	In vitro: Murine 6-3 microglia	1 µM	48 h	IFN-γ (50 U/mL)	= MTT absorbance	= MTT absorbance	↑NO	= NO	–	–
		10 µM	48 h	IFN-γ (50 U/mL)	= MTT absorbance, ↑[Ca^2+^]	= MTT absorbance, =[Ca^2+^]i	↑NO	= NO	–	–

SSRI: selective serotonin re-uptake inhibitor; SNRI: serotonin and norepinephrine re-uptake inhibitors; LPS: lipopolysaccharide; INF: interferon; IL: interleukin; mRNA: messenger RNA; NO: nitric oxide; NOS: nitric oxide synthase; TNF: tumour necrosis factor; NF-κB: nuclear factor kappa light-chain enhancer of activated B cell↑: significant increase; ↓: significant decrease; =: no significant change.

In addition, Su et al. (2015) investigated the effect of fluoxetine on microglial activation upon treatment with both IFN-γ and LPS. They showed that fluoxetine was able to prevent the increased expression of the M1 microglial surface marker CD68 protein in both rat primary and mice BV2 microglia cells. Moreover, in mice BV2 microglia, both 20 and 60 µM concentrations of fluoxetine prevented IFN-γ and LPS- induced expression of *IL-6*, *IL-1β* and *TNF-α* genes, but not of IL-1β and TNF-α proteins. Whereas, in rat primary microglia, while both concentrations of fluoxetine prevented IFN-γ- and LPS-induced increase in the expression of *IL-6* gene and IL-1β protein, only 60 µM fluoxetine prevented the increase in the expression of *IL-1β* and *TNF-α* genes. Moreover, both concentrations of fluoxetine prevented IFN-γ- and LPS-induced *iNOS* gene expression in both cell types (Su et al., 2015).

##### Paroxetine

Only one in vitro study, previously mentioned, investigated the effect of paroxetine on IFN-γ-induced microglial activation (Horikawa et al., 2010) (Table 2). In their study, Horikawa et al. (2010) showed that paroxetine did not affect cell viability and IL-4 production, and that only concentrations of paroxetine equal to 5 µM were able to prevent IFN-γ-induced intracellular Ca^2+^, TNF-α release and NO production in murine 6-3 microglial cells.

##### Citalopram

Only one in vitro study, previously mentioned, investigated the effects of citalopram on cytokines-induced microglia activation (Su et al., 2015; Table 2). They showed that 60 μM of S enantiomer of citalopram (S-citalopram) prevented IL-4-induced increase in CD206 protein expression in rat primary microglia cells. In addition, while 20 µM S-citalopram also prevented IL-4-induced IL-10 production, in rat primary microglia cells, 60 µM S-citalopram further increased *IL-10* gene expression and IL-10 protein expression, respectively, in rat primary microglia cells, and both in mice BV2 and primary microglial cells (Su et al., 2015).

In addition, Su et al. (2015) investigated the effect of S-citalopram on microglial activation upon treatment with both IFN-γ and LPS. They showed that fluoxetine was able to prevent the increased expression of CD68 protein in both rat primary and mice BV2 microglia cells. Moreover, in mice BV2 microglia, 20 µM S-citalopram was not able to prevent IFN-γ and LPS- induced expression of inflammatory biomarkers, while 60 µM S-citalopram was able to prevent the increased expression of *IL-6* and *TNF-α* genes and of IL-1β protein. Whereas, in rat primary microglia, while both concentrations of S-citalopram prevented IFN-γ and LPS- induced increase in the expression of *IL-6* and *IL-1β* genes and IL-1β protein, only 60 µM S-citalopram prevented the increase in the expression of *IL-1β* and *TNF-α* genes. Moreover, while both concentrations of S-citalopram prevented IFN-γ- and LPS-induced *iNOS* gene expression in rat primary microglia cells, only 60 µM S-citalopram prevented *iNOS* gene expression in mice BV2 microglia cells (Su et al., 2015).

##### Escitalopram

To our knowledge, only one ex vivo study examined the effect of escitalopram on cytokine-induced microglial activation (Wang et al., 2018a) (Table 2). In particular, Wang et al. (2018a) showed that escitalopram prevented the expression of IbA1 within the dorsal raphe nucleus of IFN-α-treated C57BL/6 J mice.

##### Sertraline

One previously mentioned study (Horikawa et al., 2010) and another study (Lu et al., 2019), both in vitro, investigated the effect of sertraline upon cytokines-induced microglial activation. Overall, the studies showed that specific concentrations of sertraline prevented immune activation and oxidative stress production upon in vitro exposure to either TNF-α or IFN-γ.

Horikawa et al. (2010) showed that only 5 µM sertraline was able to prevent IFN-γ-induced increase in the levels of intracellular Ca^2+^ in murine 6-3 microglia cells. Lu et al. (2019) showed that sertraline prevented TNF-α-induced increase in IbA1 expression in mice BV2 microglia cells. In addition, Lu et al. (2019) showed that only concentrations of sertraline equal to 1 µM prevented TNF-α-induced NF-κB protein expression. Horikawa et al. (2010), showed that 5 µM sertraline prevented the IFN-γ-induced TNF-α, but not IL-4, production in murine 6-3 microglia cells. Moreover, sertraline prevented IFN-γ-induced iNOS protein levels (Lu et al., 2019) and NO production (Horikawa et al., 2010).

##### Fluvoxamine

One in vitro study investigated the effect of fluvoxamine on IFN-γ-induced microglial activation (Hashioka et al., 2007). Specifically, Hashioka et al. (2007) showed that fluvoxamine prevented IFN-γ-induced increase in IL-6 and NO production in murine 6-3 microglia cells. Moreover, this study showed that the effect of fluvoxamine was prevented by treatment with either a cyclic adenosine monophosphate (cAMP) inhibitor or a protein kinase A (PKA) inhibitor (Hashioka et al., 2007).

#### Tricyclic antidepressants

##### Imipramine

One previously mentioned in vitro study, also showed that imipramine prevented IFN-γ-induced microglial activation (Hashioka et al., 2007). Specifically, Hashioka et al. (2007) showed that only a concentration of imipramine equal to 50 and 100 µM, but not 10 µM, prevented the IFN-γ-induced production of IL-6 and NO in murine 6-3 microglia cells. Moreover, this study showed that the effect of imipramine was prevented by treatment with either the cAMP inhibitor or the PKA inhibitor (Hashioka et al., 2007).

#### Norepinephrine reuptake inhibitors

##### Reboxetine

One previously mentioned in vitro study, also showed that reboxetine prevented IFN-γ-induced microglial activation (Hashioka et al., 2007). In their study, Hashioka et al. (2007) showed that only a concentration of reboxetine equal to 50 and 100 µM, but not 10 µM, was able to prevent IFN-γ-induced IL-6 and NO production in murine 6-3 microglia cells. Moreover, this study showed that the effect of reboxetine on IL-6 production was prevented by treatment with the cAMP inhibitor, whereas both the cAMP inhibitor and the PKA inhibitor prevented the effects of reboxetine on NO production (Hashioka et al., 2007).

#### Atypical antidepressants

##### Bupropion and agomelatine

Only one previously mentioned in vitro study also showed that both bupropion and agomelatine did not prevent IFN-γ-induced NO production in murine 6-3 microglia cells microglial activation (Horikawa et al., 2010).

## Discussion

**Figure 1.**
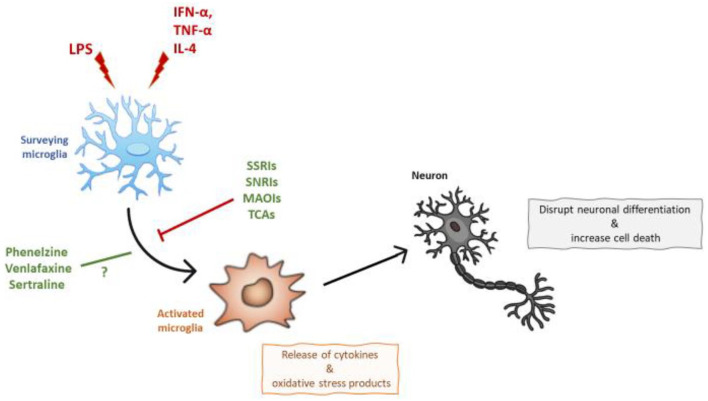
Microglial activation is induced by LPS and by IFN-α, TNF-α and IL-4, leading to neurodegeneration. As outlined in the text, this effect is prevented by SSRIs, SNRIs, MAOIs, and TCAs antidepressants.

This is the first review summarising the effects of multiple classes of antidepressants, including SSRIs, SNRIs, TCAs, MAOIs and atypical antidepressants, on microglial activation, in in vitro LPS and cytokines models of neuroinflammation. Overall, studies showed that SSRIs, SNRIs, MAOIs and TCAs antidepressants prevented microglial activation, including reduced microglial reactivity and decreased immune and oxidative stress products, in both models of LPS and cytokines (Figure 1). However, these effects were observed only for specific concentrations of the antidepressant sertraline (SSRI) in both LPS and cytokines models, and venlafaxine (SNRI) in LPS models, whereas contrasting or no effects were observed in presence of phenelzine (MAOI) in LPS models, and bupropion and agomelatine (atypical antidepressants) in cytokines models, respectively.

First of all, the majority of the studies discussed in this review showed that all classes of antidepressants were able to prevent LPS-induced cytokines release, including IL-1β, TNF-α and IL-6 (Dhami et al., 2013, 2019; Du et al., 2014, 2016; Gong et al., 2019; Liu et al., 2011, 2014; Obuchowicz et al., 2006; Park et al., 2019, 2020; Rodrigues et al., 2018; Tynan et al., 2012; Yang et al., 2014; Zhang et al., 2012). In addition, these antidepressants reduced cytokines production via inhibiting the activation of microglial immune-related intracellular signalling pathways, such as p38 MAPK, ERK, JNK and NF-κB (Du et al., 2014; Liu et al., 2011, 2014; Park et al., 2019, 2020; Rodrigues et al., 2018; Yang et al., 2014; Zhang et al., 2012). This is in line with evidence suggesting the ability of antidepressants to exert anti-inflammatory properties. This includes findings coming from in vivo studies using LPS and antidepressant treatment in animal models of depression (Taniguti et al., 2019), or from clinical studies using antidepressant treatment in depressed patients with sub-chronic levels of inflammation (Cattaneo et al., 2020; Kubera et al., 2001; Nazimek et al., 2017; Szałach et al., 2019). These studies identified a reduction in cytokines production, such as IL-1β, TNF-α and IL-6 in depressed patients (Cattaneo et al., 2020), similar to findings discussed in this review, therefore, suggesting that inhibition of microglia-induced immune activation might be one of the mechanisms through which antidepressants work in the brain.

Furthermore, all classes of antidepressants, except for the SSRI fluvoxamine, were able to prevent the release of NO and reactive oxygen species induced by LPS (Chung et al., 2012; Dhami et al., 2013, 2019; Du et al., 2014; Dubovický et al., 2014; Horikawa et al., 2010; Kim et al., 2018; Liu et al., 2011, 2014; Park et al., 2019, 2020; Tynan et al., 2012; Zhang et al., 2012). These results are confirmed in preclinical studies where antidepressants reduce oxidative stress production in models of depression (Krass et al., 2011; Li et al., 2006; Wegener et al., 2003), and in clinical studies of depression, where antidepressants decrease levels of serum nitrite and nitrate (Finkel et al., 1996). In particular, in the brain, reduced oxidative stress through antidepressant treatment has been shown to promote neuronal survival in models of depression (Chung et al., 2010; Kim et al., 2018). Therefore, it is likely that reducing microglial-derived production of oxidative stress products may be a mechanism through which antidepressants protect neuronal function in the brain and decrease depressive symptoms.

In addition to LPS, cytokines also can stimulate microglial activation. Our review shows that treatment with pro-inflammatory cytokines INF-γ, TNF-α or INF-α, and the SSRI fluoxetine, paroxetine, citalopram, escitalopram and sertraline can prevent microglial morphological changes, such as soma size and process length, as well as increased expression of the microglia inflammatory marker CD68 (Hashioka et al., 2007; Horikawa et al., 2010; Lu et al., 2019; Su et al., 2015; Wang et al., 2018a). These findings are supported by the previous literature, which has consistently reported that cytokine exposure can induce a pro-inflammatory phenotype, with associated M1 changes in microglia morphology (Anderson et al., 2017). Most interestingly, treatment with fluoxetine, citalopram and the anti-inflammatory cytokine IL-4 further increased the expression of the microglia surface marker CD206, which is indicative of an anti-inflammatory phenotype (Su et al., 2015). Therefore, evidence from this review seems to suggest that antidepressants can effectively change microglia status, consisting of either preventing or enhancing the effect of respectively pro-inflammatory or anti-inflammatory cytokines on these cells.

In addition to cell morphology, the SSRI fluoxetine, paroxetine, citalopram, sertraline, fluvoxamine and NRI reboxetine also prevented microglial production of downstream pro-inflammatory cytokines, such as IL-1β, TNF-α and IL-6, upon prior cellular exposure to INF-γ, TNF-α or INF-α treatment (Hashioka et al., 2007; Horikawa et al., 2010; Lu et al., 2019; Su et al., 2015). Once produced, cytokines, and particularly pro-inflammatory cytokines, can alter neurogenesis, a process potentially disrupted in depression, and required for antidepressant efficacy (Boldrini et al., 2013, 2019). Indeed, evidence coming from our lab has shown that in vitro treatment of human hippocampal neuronal progenitors with exogenous IL-1β, IL-6 or IFN-α can dramatically reduce cell proliferation and neurogenesis, and increase apoptosis (Borsini et al., 2017, 2018, 2020). Since microglia can disrupt neurogenesis via the production of inflammatory cytokines (Hickman et al., 2018), antidepressants treatment may exert its properties via inhibiting microglia-induced cytokines production and ultimately enhance brain plasticity and decrease depressive symptoms.

Similar to LPS, all antidepressants were able to prevent an increase in oxidative stress induced by treatment with pro-inflammatory cytokines. Specifically, the SSRI fluoxetine, citalopram and sertraline prevented the production of iNOS, whereas the SSRI paroxetine, sertraline, fluvoxamine and imipramine prevented the production of NO (Hashioka et al., 2007; Horikawa et al., 2010; Lu et al., 2019; Su et al., 2015). This shows that antidepressants can limit the effect of pro-inflammatory cytokines, released by other glial cells, neurons or peripheral immune cells, on microglia-mediated oxidative stress in the brain. This evidence proposes a new mechanistic route through which antidepressants may exert their concomitant anti-inflammatory and antioxidant properties.

While it is clear that most antidepressants are able to reduce microglial activation, for some others, including the SSRI sertraline, the SNRI venlafaxine and the MAOI phenelzine, the results were inconsistent. For example, sertraline prevented immune activation and oxidative stress production upon in vitro exposure to LPS and cytokines, but only upon concentrations higher than 5 µM. Venlafaxine also prevented immune activation only at concentrations higher than 100 µM, but did not have any preventive effect on oxidative stress production upon cell exposure to LPS. Interestingly, these results are in line with clinical and in vivo studies showing that higher doses of venlafaxine are associated with more efficacy (Mendels et al., 1993; Zhang et al., 2019). Microglia are known to be extremely sensitive to their environment, and small differences in chemical concentration are enough to alter their phenotype. This stresses the importance of developing more controlled experiments when investigating microglial polarisation, as even subtle experimental differences can contribute to the development of multiple microglia phenotypes. As for sertraline and venlafaxine, there was conflicting evidence for the action of the MAOI phenelzine, with one study showing that the antidepressant can prevent LPS-induced inflammation and oxidative stress (Dhami et al., 2013), and another study showing the opposite (Chung et al., 2012). Although, in this case, the concentration of the antidepressant was the same, concentrations of LPS, as well as microglia cells origin, were different, therefore, potentially explaining the contrasting findings.

Interestingly, two studies using magnetic resonance spectroscopy to determine the concentration of fluoxetine and fluvoxamine within the human brain of patients undergoing antidepressant treatment (Bolo et al., 2000; Henry et al., 2005) indicated that the brain concentration of fluoxetine and fluvoxamine, after a minimum of 3 weeks of treatment, ranged between 12 and 25 μM. Importantly, these concentrations coincide with the same concentrations at which these compounds exerted their anti-inflammatory actions within the presented studies (Dhami et al., 2013; Du et al., 2014, 2016; Park et al., 2019, 2020; Tynan et al., 2012).

Finally, only one study investigated atypical antidepressants and showed that treatment with bupropion and agomelatine neither prevented changes in microglia morphology nor in NO production by IFN-γ challenge (Horikawa et al., 2010). However, to our knowledge, it is the only study investigating the effects of atypical antidepressants on cytokines-induced microglial activation and they have used only one experimental model (murine 6-3 microglia) and one concentration of IFN-γ (100 U/mL). Further studies using a small incremental change of concentration of antidepressants as well as different cellular models will be necessary to understand more effectively how atypical antidepressants regulate microglial activation.

Finally, BV2 cells are a well-characterised, widely used model for microglia. Different studies, including complex cell–cell interaction studies (Henn et al., 2009), have verified that BV2 cells are a valid substitute for primary microglia in many experimental settings. However, there are some limitations to using these cells, including their murine origin (Timmerman et al., 2018). Hence, further studies will be needed in human iPSCs-induced microglia cells or other primary cultures of microglia to confirm that similar sets of genes are indeed expressed when compared with BV2 cells.

The concentration of antidepressants used in these studies are within a similar range as the concentrations observed in the brain after a treatment dose in humans (Nikisch et al., 2004, 2005; Paulzen et al., 2015); therefore, our results support microglial activation as one key pharmacological mechanism behind antidepressant action. Moreover, while this review has limitations including differences in the concentrations of antidepressants, LPS and cytokines used, in the cellular models employed, as well as in the heterogeneity of the method used to quantify cytokines production (e.g. enzyme-linked immunosorbent assay (ELISA), Western blotting analysis and reverse transcriptase polymerase chain reaction (RT-PCR)), this is the first attempt ever made to summarise both in vitro and ex vivo studies investigating the effects of different classes of antidepressants on microglial activation, by examining cellular changes and/or via measuring the production of immune and/or oxidative stress signalling molecules in microglia exposed to models of neuroinflammation. Overall, our review shows that antidepressants can significantly regulate microglia phenotype, and ultimately prevent its activation, both at a cellular and molecular level. Further research is needed to better understand the role of microglia in depression; however, the evidence so far strongly suggest that microglia are effective cellular targets of the antidepressant treatment and a mean through which antidepressants may regulate both brain inflammation and oxidative stress. Finally, to better understand the mechanisms underlying microglial activation, future ex vivo studies should focus on distinct morphological changes observed by real-time imaging of process speed, especially in response to ‘injury’, or improve phenotype analyses using cell sorting via fluorescence-activated cell sorting (FACS). Moreover, most of the studies included have investigated neurogenesis as an outcome. In the future, more attention should be given to synaptic changes and plasticity.
